# Croup

**Published:** 1878-05

**Authors:** 


*Croup* is so common a disease among children that it requires no description here; it affects the windpipe. As it attacks suddenly, most often in the night, and as an hour's time may be all the difference between life and death, it is proper to state the most reliable course to be pursued *until a physician can be obtained.*

1st. Keep the feet warm by having a jug of hot water kept against them; let them also be well wrapped up in woolen flannel.

2nd. Have a bucket of water almost as hot as the hand can bear. Have two pieces of woolen flannel of several thicknesses, one being on the throat while the other is in the hot water, renew every two or three minutes, until relief is given or the physician arrives. The water in the bucket must be kept hot by the constant addition of boiling water.

